# OAT10/SLC22A13 Acts as a Renal Urate Re-Absorber: Clinico-Genetic and Functional Analyses With Pharmacological Impacts

**DOI:** 10.3389/fphar.2022.842717

**Published:** 2022-04-06

**Authors:** Yu Toyoda, Yusuke Kawamura, Akiyoshi Nakayama, Keito Morimoto, Seiko Shimizu, Yuki Tanahashi, Takashi Tamura, Takaaki Kondo, Yasufumi Kato, Kimiyoshi Ichida, Hiroshi Suzuki, Nariyoshi Shinomiya, Yasushi Kobayashi, Tappei Takada, Hirotaka Matsuo

**Affiliations:** ^1^ Department of Pharmacy, The University of Tokyo Hospital, Tokyo, Japan; ^2^ Department of Integrative Physiology and Bio-Nano Medicine, National Defense Medical College, Saitama, Japan; ^3^ Department of Preventive Medicine, Nagoya University Graduate School of Medicine, Aichi, Japan; ^4^ Program in Radiological and Medical Laboratory Sciences, Pathophysiological Laboratory Science, Nagoya University Graduate School of Medicine, Aichi, Japan; ^5^ Department of Pathophysiology, Tokyo University of Pharmacy and Life Sciences, Tokyo, Japan; ^6^ Department of Anatomy and Neurobiology, National Defense Medical College, Saitama, Japan

**Keywords:** uricosuric agent, urate reabsorption inhibitor, urate-lowering therapy, losartan, renal urate handling, lesinurad, urate transporter

## Abstract

Dysfunctional missense variant of *organic anion transporter 10* (*OAT10*/*SLC22A13*), rs117371763 (c.1129C>T; p.R377C), is associated with a lower susceptibility to gout. OAT10 is a urate transporter; however, its physiological role in urate handling remains unclear. We hypothesized that OAT10 could be a renal urate re-absorber that will be a new molecular target of urate-lowering therapy like urate transporter 1 (URAT1, a physiologically-important well-known renal urate re-absorber) and aimed to examine the effect of OAT10 dysfunction on renal urate handling. For this purpose, we conducted quantitative trait locus analyses of serum urate and fractional excretion of uric acid (FE_UA_) using samples obtained from 4,521 Japanese males. Moreover, we performed immunohistochemical and functional analyses to assess the molecular properties of OAT10 as a renal urate transporter and evaluated its potential interaction with urate-lowering drugs. Clinico-genetic analyses revealed that carriers with the dysfunctional *OAT10* variant exhibited significantly lower serum urate levels and higher FE_UA_ values than the non-carriers, indicating that dysfunction of OAT10 increases renal urate excretion. Given the results of functional assays and immunohistochemical analysis demonstrating the expression of human OAT10 in the apical side of renal proximal tubular cells, our data indicate that OAT10 is involved in the renal urate reabsorption in renal proximal tubules from urine. Additionally, we found that renal OAT10 inhibition might be involved in the urate-lowering effect of losartan and lesinurad which exhibit uricosuric effects; indeed, losartan, an approved drug, inhibits OAT10 more strongly than URAT1. Accordingly, OAT10 can be a novel potential molecular target for urate-lowering therapy.

## Introduction

Elevated serum urate levels can cause gout, the most common form of inflammatory arthritis (Dalbeth et al., 2019). Under physiological conditions, uric acid—the terminal metabolite of purine metabolism in humans—primarily exists as urate, an anion form. Because urate cannot passively permeate across cellular membranes, urate transporters play a pivotal role in urate handling in our body. To date, urate transporter 1 (URAT1/SLC22A12) (Enomoto et al., 2002; Ichida et al., 2004), glucose transporter 9 (GLUT9/SLC2A9) (Matsuo et al., 2008; Dinour et al., 2010), and ATP-binding cassette transporter G2 (ABCG2/BCRP) (Matsuo et al., 2009; Woodward et al., 2009) have been identified as physiologically important urate transporters of which genetic dysfunction influences serum urate levels; therefore, these three transporters have been extensively studied in the context of urate regulation in our body. Nevertheless, such urate transporters do not provide a comprehensive picture of urate-handling systems which are also related to the risk of gout/hyperuricemia in humans. Besides, this point is supported by previous findings in animal studies. For instances, *Abcg2* knockout mice retain approximately half levels of intestinal urate secretion activities compared with wild-type mice (Ichida et al., 2012); genetic disruption of *Urat1* in *uricase* knockout mice results in the elevation of fractional excretion of uric acid (FE_UA_) (around to 50%, mean value) but not reaching to 100% (Hosoyamada et al., 2016), indicating the presence of other latent machineries that would be theoretically involved in the remaining portions of urate transport.

The kidney is an important organ for regulating serum urate levels by re-absorbing almost all of filtered urate and is responsible for two-thirds of the net urate secretion from the body. Regarding the renal urate re-absorption route, both URAT1 (as an importer) and GLUT9 (as an exporter) form a cascaded system of transcellular urate transport from urine to blood *via* renal proximal tubular cells as apical (urine side) and basal (blood side) machinery, respectively. It is noteworthy that this route remains functional to some extent even in subjects genetically lacking *URAT1* (Ichida et al., 2004) as well as in animal models with *Urat1* knockout genetic background (Hosoyamada et al., 2010; Hosoyamada et al., 2016), suggesting that there is an alternative to URAT1. Hitherto, based on urate transport activities determined by *in vitro* experiments and renal expression, several transporters have been proposed as the candidates including SLC22 family proteins (Koepsell, 2013; Halperin Kuhns and Woodward, 2021). Among them, organic anion transporter 10 (OAT10)/SLC22A13 is strongly expressed in the kidney and transport assays using *Xenopus* oocytes (Bahn et al., 2008) and mammalian culture cells (Higashino et al., 2020) demonstrated that urate is an OAT10 substrate. However, not only affinity of OAT10 protein to urate but also the effects of OAT10 dysfunction on the renal urate handling remain unclear.

In this study, we hypothesized that OAT10 could be a physiologically important machinery involved in the renal urate re-absorption based on our recent study (Higashino et al., 2020), in which clinico-genetic and functional studies revealed that a functionally null variant of *OAT10* (c.1129C>T; p.R377C that diminishes the urate transport activity of OAT10 with little effect on its cellular protein level and plasma membrane localization) significantly decreases the risk of gout (odds ratio, OR = 0.67). This finding, together with the lower serum urate levels associated with this dysfunctional variant (Higashino et al., 2020), led us to reason that OAT10 could have a physiological impact on renal urate handling. To clarify the proposed involvement of OAT10 in renal urate re-absorption that remains speculation for a long time, we herein investigated the physiological role of OAT10 *via* a clinico-genetic approach. Moreover, to gain insight into the molecular properties of OAT10 as a urate transporter, including interaction with various urate-lowering drugs, we conducted functional analyses.

## Materials and Methods

### Ethics Approval

The studies involving human participants were reviewed and approved by the Institutional Ethics Committees of the National Defense Medical College and Nagoya University. All protocols were in accordance with the Declaration of Helsinki, and written informed consent was obtained from each participant in the present study.

### Participants

A total of 4,521 Japanese men were recruited from health examination participants in Shizuoka and Daiko areas of the Japan Multi-Institutional Collaborative Cohort Study (Hamajima, 2007; Asai et al., 2009). The characteristics of the participants are shown in Table 1. Of note, under normal physiological conditions, uric acid (molecular weight of 168.1) exists in the blood mainly as urate (the anion form of uric acid, molecular weight of 167.1); on the other hand, in clinical testing method, this parameter is measured and converted to concentration of “uric acid (mg/dl)” in the serum, as described previously (Nakatochi et al., 2021). Nevertheless, based on the G-CAN (The Gout, Hyperuricemia, and Crystal-Associated Disease Network) nomenclature for gout (Bursill et al., 2019), we herein used the term of “serum urate,” instead of serum uric acid, for mentioning the circulating form.

**TABLE 1:** Characteristics of the study participants.

	Number	Age (year)	Body-mass index (kg/m^2^)
Participants whose FE_UA_ data were available	1,518	51.9 ± 8.39	23.3 ± 2.77
All participants^a^	4,521	52.9 ± 9.21	23.4 ± 2.89

Japanese male individuals were recruited from health examination participants in Shizuoka and Daiko areas of the Japan Multi-Institutional Collaborative Cohort Study (Hamajima, 2007; Asai et al., 2009). Data are expressed as means ± SD. FE_UA_, fractional excretion of uric acid.

a A population studied in this study did not include any subjects with hypouricemia (serum urate ≤ 2.0 mg/dl) (Nakayama et al., 2019; Kawamura et al., 2021).

Quantitative trait locus (QTL) analysis of serum urate was performed for all participants. Among them, for 1,518 men with available urinary data, we performed subsequent QTL analysis of FE_UA_. Based on the results of blood and urine tests, FE_UA_ was calculated as follows [urinary urate (mg/dl) × serum creatinine (mg/dl)]/[serum urate (mg/dl) × urinary creatinine (mg/dl)] × 100 (%).

### Genetic Analysis of Human *OAT10*/*SLC22A13* Gene

Genomic DNA was extracted from whole peripheral blood cells of the 4,521 Japanese male participants according to methods described in our previous study (Sakiyama et al., 2021). To genotype rs117371763 (c.1129C>T: p.R377C) of the *OAT10*/*SLC22A13* gene, we employed the TaqMan method (Thermo Fisher Scientific, Kanagawa, Japan) using a QuantStudio 5 real-time PCR system (Thermo Fisher Scientific), as described in our previous study (Higashino et al., 2020). Also, like in the previous study, to confirm the accuracy of the genotyping results, >100 samples were also subjected to direct sequencing with the following primers: 5′-TGG​TGT​GTG​TTG​GCA​GAG-3′ and 5′-GGT​CCC​ATC​CAC​TGG​AAC-3′. DNA sequence analysis was performed with a 3130xl Genetic Analyzer (Thermo Fisher Scientific). Genotype distributions were as follows: 1129C/C, 4,128 subjects; 1129C/T, 384 subjects; 1129T/T, 9 subjects. Among them, there were 1,369, 143, and 6 subjects with available urinary data, respectively.

### Materials

The critical materials and resources used in this study were summarized in Supplementary Table S1. Dotinurad was kindly provided by Fuji Yakuhin (Saitama, Japan) under a material transfer agreement. All other chemicals used were commercially available and of analytical grade. In this study, we evaluated the following urate-lowering drugs and their active metabolites: urate synthesis inhibitors (allopurinol, oxypurinol, febuxostat, and topiroxostat), uricosuric agents (benzbromarone, 6-hydroxybenzbromarone, dotinurad, lesinurad, and probenecid), and other drugs that decrease serum urate levels with an action mechanism different from their expected medicinal effects (fenofibrate and losartan). Detailed information is summarized in Table 2.

**TABLE 2:** List of urate-lowering drugs and their reactive metabolites used in the present study.

Urate-lowering drugs	(μM)^a^	References for urate-lowering effect
Urate synthesis inhibitors		
Allopurinol	100	Watts et al. (1965)
Oxypurinol	100	Watts et al. (1965)
Febuxostat	10	Osada et al. (1993)
Topiroxostat	10	Okamoto et al. (2003)
Uricosuric agents		
Benzbromarone	100	Enomoto et al. (2002)
6-Hydroxybenzbromarone	100	Oikawa et al. (2005)
Dotinurad	100	Taniguchi et al. (2019)
Lesinurad	100	Miner et al. (2016)
Probenecid	100	Gutman et al. (1954)
Other drugs^b^		
Fenofibrate	100	Harvengt et al. (1980)
Losartan	100	Kamper and Nielsen. (2001)

a Maximum concentrations used in the present study.

b These two drugs unintentionally (with an action mechanism different from their expected medicinal effects) decrease serum urate levels.

### Generation of an Anti-OAT10 Polyclonal Antibody

An anti-human organic anion transporter 10 (OAT10) polyclonal antibody was raised in rabbits according to methods described in our previous study (Takada et al., 2015). In brief, the rabbit polyclonal antibody was generated against two KLH-coupled human OAT10 peptides as follows: amino acids ^#^523–536 (KLH)-C + PKSVPSEKETEAKG; amino acids ^#^293–306, (KLH)-C + KAASVNRRKLSPEL, which were also used to determine the titer of antiserum. On day 77, whole blood was collected from an adequately-immunized rabbit. The prepared antiserum was further purified before use using an epitopes-conjugated affinity column.

### Immunohistochemistry

Immunohistochemical analysis using the anti-OAT10 antibody was conducted according to our previous study (Chiba et al., 2015). The analyzed tissue was the normal part of the human kidney obtained from a male patient with renal cell carcinoma. In brief, with 4-μm paraffin sections of the kidney, antigen retrieval was conducted by microwave oven-heating in a retrieval solution (0.01 M citrate buffer, pH 6.0) for 10 min; followed by inactivation of endogenous peroxidase activity with hydrogen peroxide (3% in methanol) treatment for 10 min, and further washing with Tris-buffered saline containing 0.05% Tween 20 (TBST). After blocking with 5% goat serum in TBS for 10 min, the sections were incubated with the anti-OAT10 antibody (1:50 dilution in TBS containing 1% bovine serum albumin, BSA) at 4°C overnight; subsequently the sections were treated with the Dako EnVision™ Kit/horseradish peroxidase (HRP) (K1491; Dako, Kyoto, Japan) as a secondary antibody, followed by 3,3′-diaminobenzidine (Dako) treatment for the detection of the immune reactions. Nuclei were counterstained with Mayer’s hematoxylin. For antigen absorption test, to block specific staining, the anti-OAT10 antibody was pre-incubated with the OAT10 peptides, which were used at a concentration of 100 μg/ml, before staining procedure. After visualization, the specimens were imaged using an upright microscope (Eclipse 50i; Nikon, Tokyo, Japan) equipped with a Nikon Digital Sight DS-5M camera (Nikon).

### Plasmid Construction

The full-length human OAT10/SLC22A13 wild-type (WT) (NCBI accession; NM_004256) open reading frame (ORF) in pEGFP-N1 plasmid was constructed in our previous study (Higashino et al., 2020). The full-length human URAT1/SLC22A12 WT (NCBI accession; NM_144585.3) ORF in pEGFP-C1 was derived from our previous study (Toyoda et al., 2020a). Prior to further experiments described below, each plasmid containing the transporter and control empty vectors were obtained in the same lot using a PureLink™ HiPure Plasmid Filter Midiprep Kit (Thermo Fisher Scientific).

### Cell Culture

Human embryonic kidney 293 (HEK293)-derived 293A cells were maintained in Dulbecco’s Modified Eagle’s Medium (DMEM) (Nacalai Tesque, Kyoto, Japan) supplemented with 10% fetal bovine serum (Biowest, Nuaillé, France), 1% penicillin-streptomycin (Nacalai Tesque), 2 mM L-glutamine (Nacalai Tesque), and 1 × Non-Essential Amino Acid (Life Technologies, Tokyo, Japan) at 37°C in a humidified atmosphere of 5% (v/v) CO_2_ in air; we confirmed that 293A cells used were negative for mycoplasma contamination using MycoAlert™ mycoplasma detection kit (Lonza, Basel, Switzerland). Madin-Darby canine kidney II (MDCKII) cells (a well-used polarized renal cell line to investigate the cellular localization of transporter proteins—apical or basal membranes) were also maintained in a similar way.

For the *in vitro* transport assay, 24 h after the seeding of the cells (0.92 × 10^5^ cells/cm^2^) onto 12-well cell culture plates, each vector plasmid was transfected into 293A cells with a forward transfection approach using polyethyleneimine “MAX” (PEI-MAX) (Polysciences, Warrington, PA, United States) as described previously (Toyoda et al., 2020a). All experiments were carried out with 293A cells at passages 12–20. Of note, such mammalian cell lines have been well-used to evaluate urate transport activity of target protein (Miner et al., 2016; Toyoda et al., 2020b; Higashino et al., 2020).

For confocal microscopy, each vector plasmid was transfected into MDCKII cells with a reverse transfection approach using PEI-MAX as described previously (Toyoda et al., 2016). In brief, MDCKII cells were collected after trypsinization, followed by centrifugation at 1,000 × *g* for 5 min. The cell pellet was re-suspended in fresh DMEM, and the resulting suspension was mixed with plasmid/PEI-MAX mixture (2 μg of plasmid/10 μl of PEI-MAX for 1 × 10^6^ cells of MDCKII cells). Then, the MDCKII cells were re-seeded onto cell culture plates at a concentration of 1 × 10^5^ cells/cm^2^. For whole cell lysate preparations, MDCKII cells were re-seeded onto 6-well cell culture plates at the same concentration. All experiments were carried out using MDCKII cells at passages 7–10.

### Preparation of Protein Lysates and Immunoblotting

After preparation of whole cell lysates with cell lysis buffer A [50 mM Tris/HCl (pH 7.4), 1 mM dithiothreitol, 1% (v/v) Triton X-100, and cOmplete™, EDTA-free protease inhibitor cocktail (Roche, Basel, Switzerland)], to examine the *N*-linked glycosylation status of OAT10 and URAT1 proteins, whole cell lysate samples were treated with Peptide *N*-glycosidase F (PNGase F) (New England Biolabs, Ipswich, MA, United States) as described previously (Higashino et al., 2020). The protein concentration was determined using the Pierce™ BCA Protein Assay Kit (Thermo Fisher Scientific) with bovine serum albumin (BSA) as a standard according to the manufacturer’s protocol. The samples were separated using SDS-PAGE and transferred to an Immobilon-P PVDF membrane (Millipore, Bedford, MA, United States) by electroblotting at 15 V for 60 min. Blots were probed with appropriate antibodies (Supplementary Table S1) according to previous studies (Toyoda et al., 2020a; Higashino et al., 2020), and the signals were visualized by using chemiluminescence and detected using a multi-imaging analyzer Fusion Solo 4™ system (Vilber Lourmat, Eberhardzell, Germany).

### Confocal Microscopic Observation

Confocal laser scanning microscopic observation was conducted as described previously, with some minor modifications (Toyoda et al., 2016). In brief, 72 h after the transfection, MDCKII cells were fixed with ice-cold methanol for 10 min at room temperature. After washing three times with PBS (−) containing 1% BSA (BSA-PBS), the cells were incubated with a mouse anti-gp135 monoclonal antibody (3F2/D8; the Developmental Studies Hybridoma Bank, Iowa City, IA, United States) diluted 100-fold in BSA-PBS for 1 h at room temperature, followed by incubation with a goat anti-mouse IgG-Alexa Fluor 546-conjugate (A-11003; Thermo Fisher Scientific), diluted 200-fold in BSA-PBS, for 1 h at room temperature in the dark. Subsequently, cells were subjected to TO-PRO-3 Iodide (Molecular Probes, Eugene, OR, United States) staining. After the visualization of the nuclei, the cells were mounted in VECTASHIELD Mounting Medium (Vector Laboratories, Burlingame, CA, United States). To analyze the localization of EGFP-fused OAT10 or EGFP-fused URAT1 proteins, fluorescence was detected using an FV10i Confocal Laser Scanning Microscope (Olympus, Tokyo, Japan).

### Urate Uptake Assay

With OAT10, urate uptake assay using OAT10-expressing 293A cells was conducted according to our previous study (Higashino et al., 2020). In brief, 48 h after the plasmid transfection, the cells were washed twice with a transport buffer (Ringer solution: 130 mM NaCl, 4 mM KCl, 1 mM Na_2_HPO_4_, 1 mM MgSO_4_, 1 mM CaCl_2_, 20 mM HEPES, 18 mM D-glucose, at pH 6.4 unless otherwise indicated) and pre-incubated in Ringer solution for 10 min at 37°C. The cells were further incubated for 1 min in pre-warmed fresh Ringer solution containing 10 μM [8–^14^C]-urate unless otherwise indicated. To examine the OAT10-inhibitory effects of the target compounds, Ringer solution either without (i.e., with only vehicle control, 0.1% dimethyl sulfoxide, DMSO) or with the individual compounds at the indicated concentrations was used. Subsequently, the cells were washed three times with ice-cold Ringer solution, lysed with 500 μl of 0.2 M NaOH, and neutralized with 100 μl of 1 M HCl. We then measured the radioactivity in the lysate using a liquid scintillator (Tri-Carb 3110TR; PerkinElmer, Waltham, MA, United States). Protein concentrations were determined using the Pierce™ BCA Protein Assay Kit, as described above. The urate transport activity was calculated as the incorporated clearance (μL/mg protein/min) [incorporated level of urate (DPM/mg protein/min)/urate level in the incubation mixture (DPM/μL)]. The OAT10-mediated urate transport activity was calculated by subtracting the urate transport activity of mock cells from that of OAT10-expressing cells. To examine the OAT10-inhibitory effects of the target compounds, their effects on the urate transport activity of mock cells were also examined.

With URAT1, urate uptake assay using URAT1-expressing 293A cells was conducted with a Cl^−^-free transport buffer [Cl^−^-free Hanks’ Balanced Salt Solution (HBSS): 125 mM Na-gluconate, 4.8 mM K-gluconate, 1.2 mM KH_2_PO_4_, 1.2 mM MgSO_4_, 1.3 mM Ca-gluconate, 25 mM HEPES, 5.6 mM D-glucose, at pH 6.4 unless otherwise indicated] in a similar way according to our previous studies (Miyata et al., 2016; Toyoda et al., 2020a). In this case, after pre-incubation in Cl^−^-free HBSS for 15 min at 37°C, the cells were further incubated for 20 s in pre-warmed fresh Cl^−^-free HBSS containing 10 μM [8–^14^C]-urate either without (i.e*.*, with only vehicle control, 0.1% DMSO) or with the individual compounds at the indicated concentrations. Of note, imposing a Cl^−^ gradient by removal of external Cl^−^ results in the acceleration of URAT1-mediated urate uptake into the cell (Enomoto et al., 2002), bringing a relative reduction in the background levels for urate transport activities. Given this advantage for conducting *in vitro* assays, the Cl^−^-free condition has been used for functional investigations on URAT1 in various studies (Anzai et al., 2004; Miyata et al., 2016; Bao et al., 2019; Toyoda et al., 2020a). In this point, we had confirmed that the Cl^−^-free HBSS is a better choice for inhibitory tests against URAT1-mediated urate transport than Ringer solution (Supplementary Figure S1).

### Calculation of the Half-Maximal Inhibitory Concentration Values

To calculate the half maximal inhibitory concentration (IC_50_) values of the target compounds against urate transport by OAT10 or URAT1, the urate transport activities were measured in the presence of each compound at several concentrations. The OAT10- or URAT1-mediated transport activities were expressed as a percentage of the control (100%). Based on the calculated values, fitting curves were obtained according to the following formula using the least-squares method with Excel 2019 (Microsoft, Redmond, WA, United States) as described previously (Toyoda et al., 2020a):

Predicted value[%]=100−(Emax×CnEC50n+Cn)

where, E_max_ is the maximum effect, EC_50_ is the half-maximal effective concentration, C is the concentration of the test compound, and n is the sigmoid-fit factor. Finally, based on these results, the IC_50_ was calculated.

### Statistics

With clinical analyses, statistical analyses were performed using SPSS v.22.0J (IBM Japan, Tokyo, Japan). Univariate linear regression analysis was used for quantitative trait locus analyses. With functional analyses, all statistical analyses were performed using Excel 2019 with Statcel4 add-in software (OMS publishing, Saitama, Japan). To determine the kinetic parameters of the OAT10-mediated urate transport—Michaelis-Menten constant (*K*_m_) and maximal velocity (*V*_max_), the Michaelis–Menten model was fitted to the experimental values of the urate transport rates, and the urate concentrations by non-linear regression curve fitting using GraphPad Prism 8 (GraphPad Software, San Diego, CA, United States). Different statistical tests were used for different experiments, as described in the table captions of figure legends which include the number of biological replicates (*n*). In the case of a single pair of quantitative data, after comparing the variances of a set of data using an *F*-test, an unpaired Student’s *t*-test was performed. Statistical significance was defined in terms of *p* values less than 0.05 or 0.01.

## Results

### Increase of Net Renal Urate Secretion Associated With the Genetic Dysfunction of *OAT10*

**FIGURE 1:**
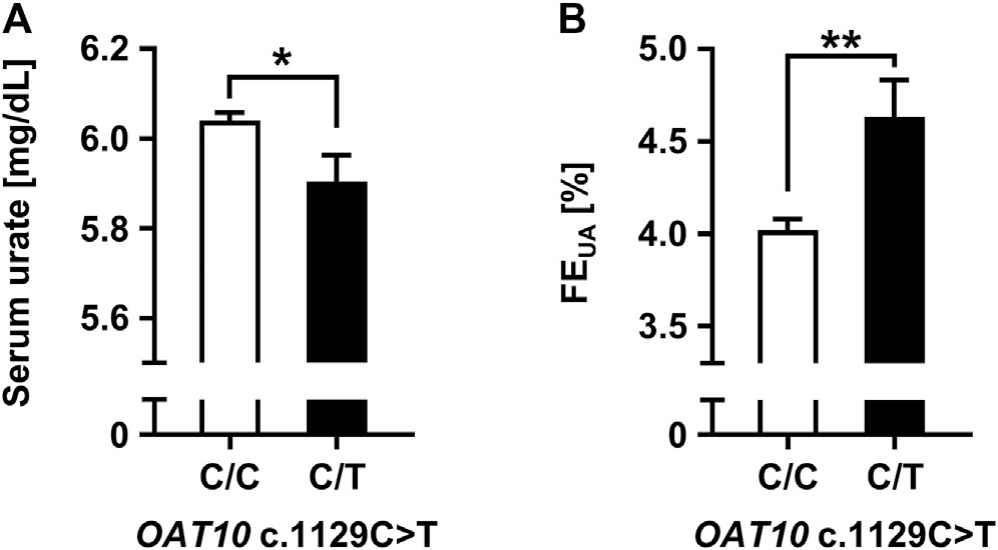
Increased renal urate excretion associated with genetic dysfunction of *OAT10*. Effects of genetic dysfunction of *OAT10* on the renal urate handling in humans; serum urate levels **(A)**, the values of fractional excretion of uric acid (FE_UA_) **(B)**. Data are expressed as the mean ± SEM. *p < 0.05; **p < 0.01 (univariate linear regression analysis).

**FIGURE 2:**
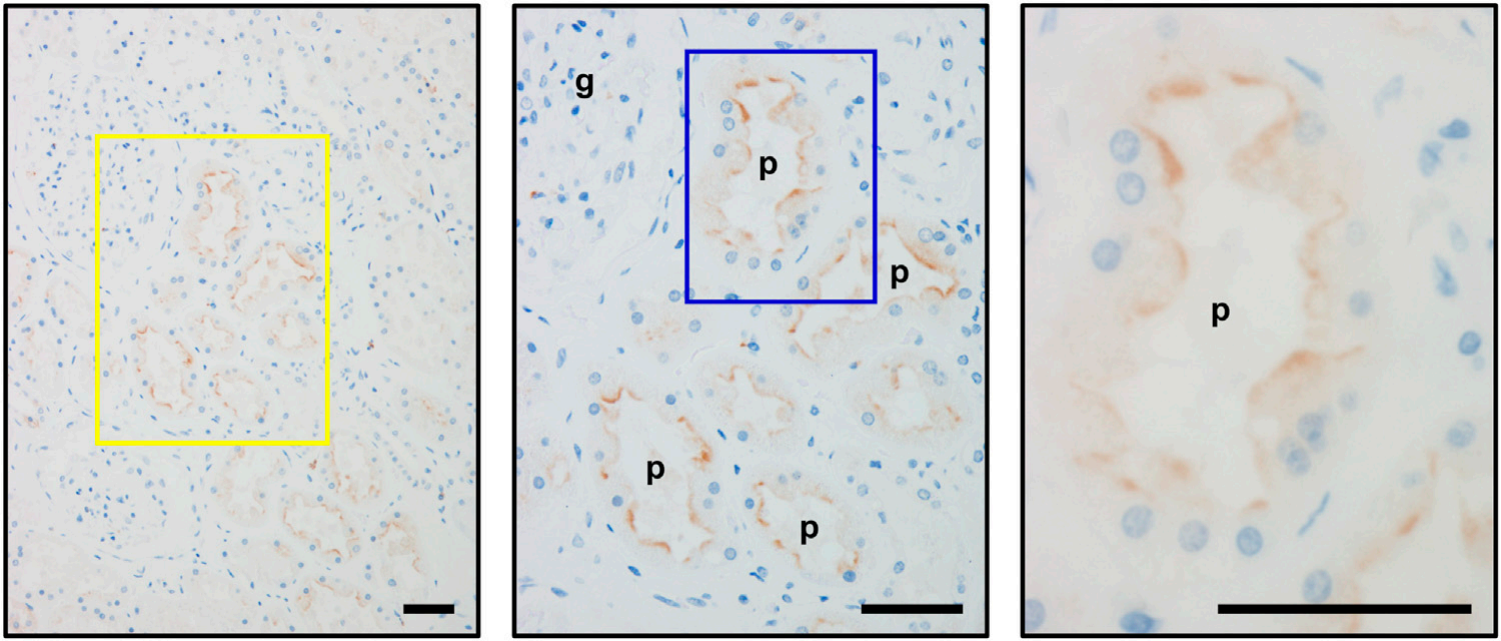
Immunohistochemical detection of OAT10 on the luminal membranes of the renal proximal tubules. Representative immunohistochemical micrographs of fixed sections of the human kidney, indicating the luminal expression of OAT10 in renal proximal tubular cells. Middle and right panels, the magnified image of the representative area outlined by a yellow and a blue rectangle, respectively. Bars, 50 μm; g, glomerulus; p, proximal tubule.

**FIGURE 3:**
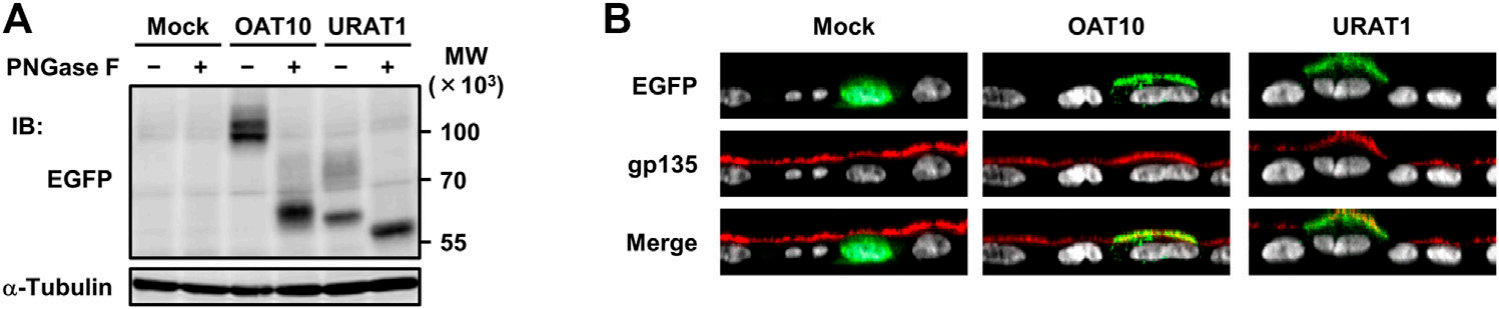
Expression and apical localization of OAT10 in polarized MDCKII cells. Immunoblot detection of OAT10 and URAT1 in whole cell lysate samples prepared from MDCKII cells 72 h after transfection **(A)**. OAT10 or URAT1 fused with EGFP was detected using an anti-EGFP antibody. PNGase F sensitive signals indicate that the target proteins were maturated as glycoproteins; α-tubulin, loading control. Representative confocal microscopic Z-sectioning images **(B)**. An endogenous apical membrane marker gp135 was immunostained using an anti-gp135 antibody (red). Nuclei were stained with TO-PRO-3 iodide (gray). Mock (EGFP), a control vector.

To investigate the effect of a genetic dysfunction of OAT10 on renal urate secretion, we conducted clinico-genetic analyses. Regarding the functionally null variant (*OAT10* c.1129C>T), only a few subjects were homozygotes (1129T/T); the others were heterozygotes (1129C/T) or wild-types (1129C/C). Thus, we compared the following parameters related to renal urate handling between the latter two groups (*OAT10* 1129C/T vs*.* 1129C/C) (Figure 1). Serum urate levels were significantly lower (Figure 1A; Supplementary Table S2) and the values of FE_UA_ were significantly higher (Figure 1B; Supplementary Table S3) in heterozygous carriers of *OAT10* c.1129T (C377) than those in non-carriers. This inverse association suggested that, like URAT1, OAT10 could be involved in urate re-uptake from urine. Additionally, the value of coefficient for the effect allele with the urate-lowering effect was −0.135 mg/dl/allele (Supplementary Table S2) which was not so large but could support the physiological importance of OAT10 as discussed later (see *Discussion*). Also, similar results were obtained when we combined the homozygotes with the heterozygotes (Supplementary Tables S4, S5). Moreover, immunohistochemistry revealed the luminal expression of OAT10 in the proximal tubules in the cortex of the human kidney (Figure 2), but the expression of OAT10 was hardly detected in the medulla, which is a similar pattern to that of URAT1 (Enomoto et al., 2002). The specificity of the anti-OAT10 antibody was verified by antigen absorption test (Supplementary Figure S2). Also, this luminal expression of OAT10 in the proximal tubules is consistent with the data in the Human Protein Atlas (Uhlén et al., 2015) (https://www.proteinatlas.org/ENSG00000172940-SLC22A13/tissue/kidney#img). Besides, when expressed in polarized MDCKII cells, OAT10 was matured as a glycoprotein (Figure 3A) and localized on the apical membrane, similar to URAT1 (Figure 3B). These results indicate that OAT10 prefers to be on the apical side of renal proximal tubule cells in the kidney, the position required for the urate re-uptake from urine into the cell. These findings are consistent with a result of immunoblotting addressing renal expression of rat Oat10 in a previous study (Bahn et al., 2008). Given these clinical and experimental data, OAT10 would be a renal urate re-absorber in humans that influences serum urate levels and net amount of renal urate excretion.

### Molecular Properties of OAT10 as a Urate Transporter

**FIGURE 4:**
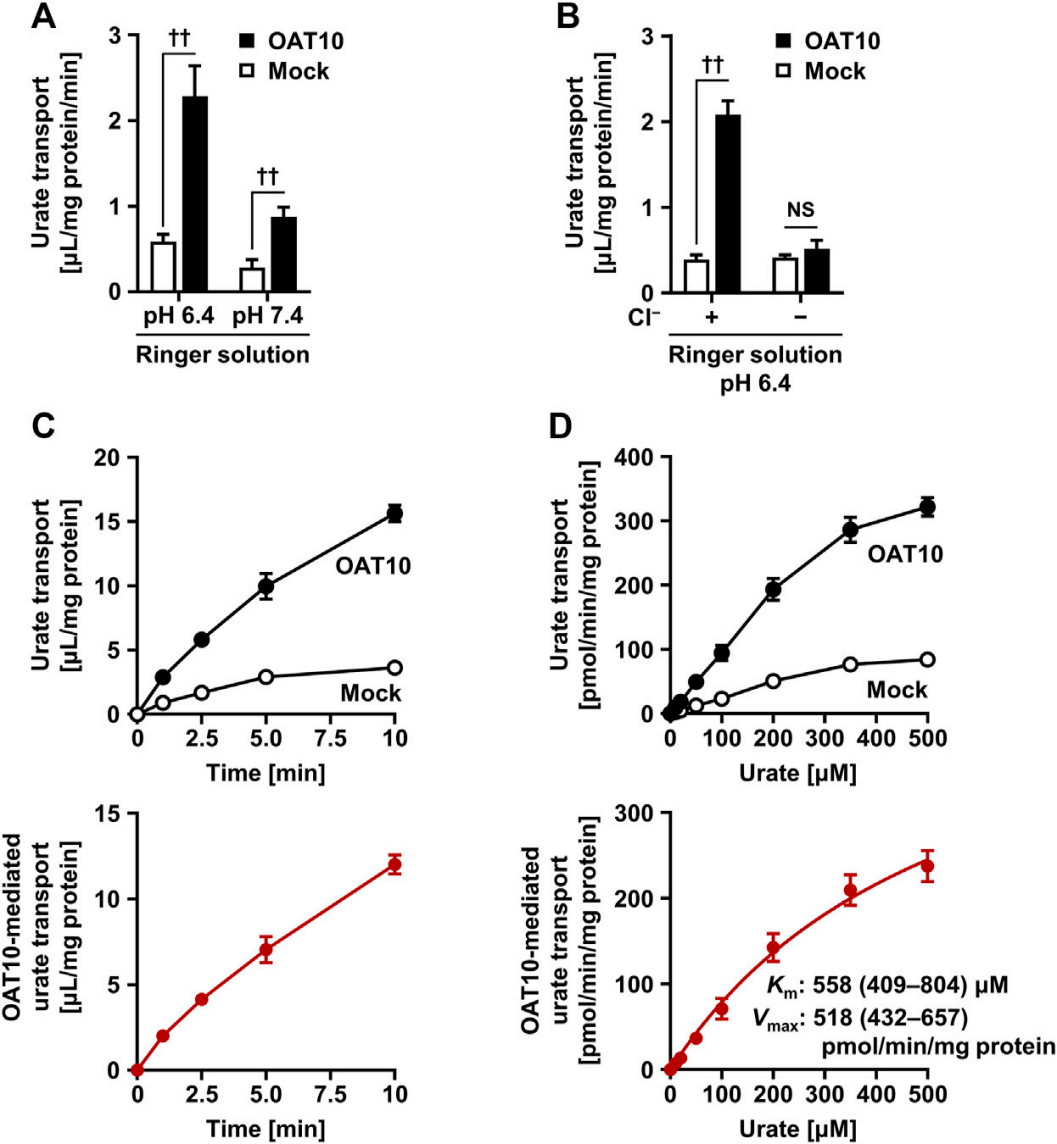
Characterization of molecular properties of OAT10 as a urate transporter. Transiently OAT10-expressing 293A cells were subjected to a cell-based urate transport assay, 48 h after transfection. Acidic pH **(A)**, external Cl^−^ **(B)**, time **(C)**, and concentration **(D)** dependent urate transport activities of OAT10 in Ringer solution (pH 6.4) containing 10 μM [8–^14^C]-urate (unless otherwise indicated). To prepare Cl^−^-free Ringer solution, all chloride salts in Ringer solution were replaced with each corresponding gluconate salt **(B)**. OAT10-mediated urate transport [lower panels: **(C,D)**] was calculated by subtracting the urate transport activity in mock cells from that in OAT10-expressing cells [upper panels: **(C,D)**]. Data are expressed as the mean ± SD; *n* = 3 **(A)**, 4 **(B–D)**. ^††^ *p* < 0.01; NS, not significantly different between groups (two-sided *t*-test). With the estimated Michaelis-Menten constant (*K*_m_) and maximal velocity (*V*_max_), the values of 95% confidence interval were in parentheses.

To gain insight into the molecular properties of OAT10 as a urate transporter, we performed *in vitro* functional analyses using transiently OAT10-expressing HEK-derived 293A cells. The measured amount of radiolabeled urate incorporated into the cells revealed that OAT10 was more active at lower pH, mimicking urine conditions (Figure 4A). A similar result was obtained in URAT1 (Supplementary Figure S1). On the other hand, in contrast to the case in URAT1, OAT10-mediated urate transport was remarkably decreased by excluding Cl^−^ from the Ringer solution (Figure 4B); the calculated OAT10-mediated urate transport activities in the Cl^−^-free condition were 6.1 ± 4.5% of those in the normal Ringer solution. This chloride-dependency is consistent with previous results obtained in an oocyte study (Mandal et al., 2017). Moreover, from time-course experiments we used uptake at 1 min to determine the initial rate of urate uptake by OAT10 (Figure 4C). Hence, uptake for 1 min at pH 6.4—a condition appropriate for the evaluation of OAT10-mediated urate transport—was examined in subsequent analyses. Under the experimentally maximum urate concentration (500 μM, which was due to the limited solubility of uric acid in the transport buffer), OAT10-mediated urate uptake was not completely saturated; thus, the estimated *K*_m_ and *V*_max_ for urate were 558 μM and 518 pmol/min per mg protein, respectively (Figure 4D). Considering that the *K*_m_ value of OAT10 was the second lowest among the already characterized human urate transporters working as importers next to URAT1 (*K*_m_ for urate, 371 μM) (Enomoto et al., 2002) (Table 3), OAT10 may have a role as a parallel backup of URAT1.

**TABLE 3:** Experimentally determined values of Michaelis-Menten constant (*K*_m_) for urate of human urate transporters expressed in the kidney.

Renal urate transporters	Localization^a^	*K*_m_ for urate (μM)	References
SLC22A family proteins			
URAT1/SLC22A12	PT, AM	371	Enomoto et al. (2002)
OAT10/SLC22A13	PT, AM	558	This paper
OAT1/SLC22A6	PT, BLM	943	Ichida et al. (2003)
OAT2/SLC22A7	PT, AM or BLM	1,168	Sato et al. (2010)
OAT3/SLC22A8	PT, BLM	2,888	Kimura et al. (2000)
OAT4/SLC22A11	PT, AM	3,780	Kimura et al. (2001)
Others			
GLUT9/SLC2A9	PT, AM and BML	890; 981	Vitart et al. (2008); Caulfield et al. (2008)
ABCG2	PT, AM	8,240	Matsuo et al. (2009)

a Information of the localization of each protein in the kidney are from a previous study (Burckhardt, 2012). URAT, urate transporter; OAT, organic anion transporter; GLUT, glucose transporter; ABCG2, ATP-binding cassette transporter G2; PT, proximal tubule; AM, apical membrane; BLM, basolateral membrane.

### Effects of Urate-Lowering Drugs and Their Reactive Metabolites on OAT10-Mediated Urate Transport

**FIGURE 5:**
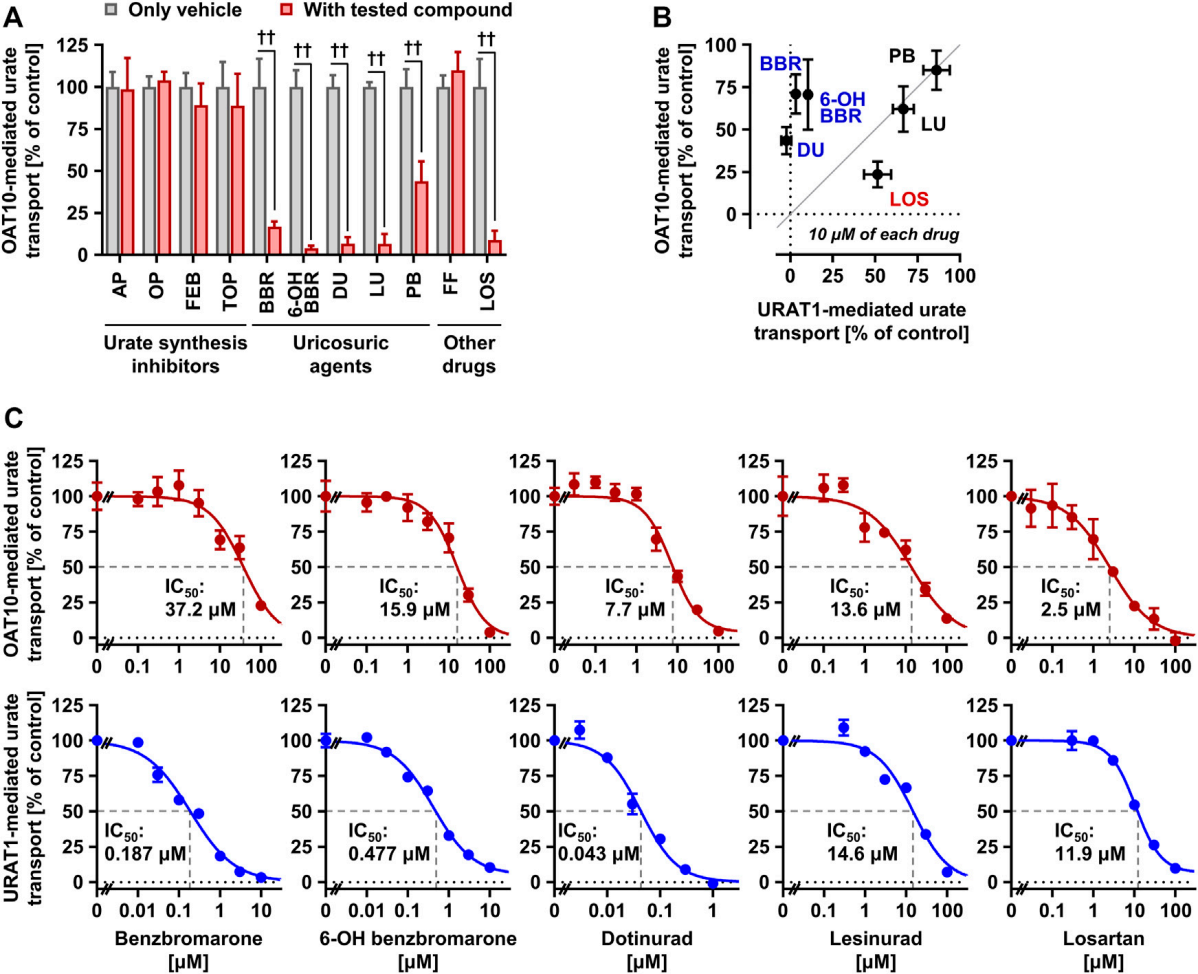
Effects of urate-lowering drugs and their reactive metabolites on the urate transport activity of OAT10**. (A)** OAT10-mediated urate transport activities in the presence or absence of urate-lowering drugs, including their reactive metabolites, at 100 μM or experimentally maximum concentrations (Table 2). AP, allopurinol; OP, oxypurinol; FEB, febuxostat; TOP, topiroxostat; BBR, benzbromarone; 6-OH BBR, 6-hydroxybenzbromarone; DU, dotinurad; LU, lesinurad; PB, probenecid; FF, fenofibrate; LOS, losartan. **(B)** Comparison of the effects of each compound (10 μM) on the urate transport activities of URAT1 and OAT10. Values are shown as % of vehicle control (only 0.1% dimethyl sulfoxide); data are expressed as the mean ± SD; *n* = 3–4 **(A,B)**. ^††^ *p* < 0.01 (two-sided *t*-test). **(C)** Concentration-dependent inhibition of OAT10-mediated urate transport by BBR, 6-OH BBR, DU, LU, and LOS. The urate transport activities were measured in the presence of each compound at indicated concentrations for 1 min. Values are shown as % of vehicle control; data are expressed as the mean ± SEM; *n* = 4.

Although some drugs used for urate-lowering therapy inhibit URAT1 function, their effects on OAT10 remain unclear. Thus, we investigated the OAT10-inhibitory activities of urate-lowering drugs and their active metabolites (total 11), including urate synthesis inhibitors, uricosuric agents, fenofibrate, and losartan, five of which were compared with those against URAT1 (Figure 5).

**FIGURE 6:**
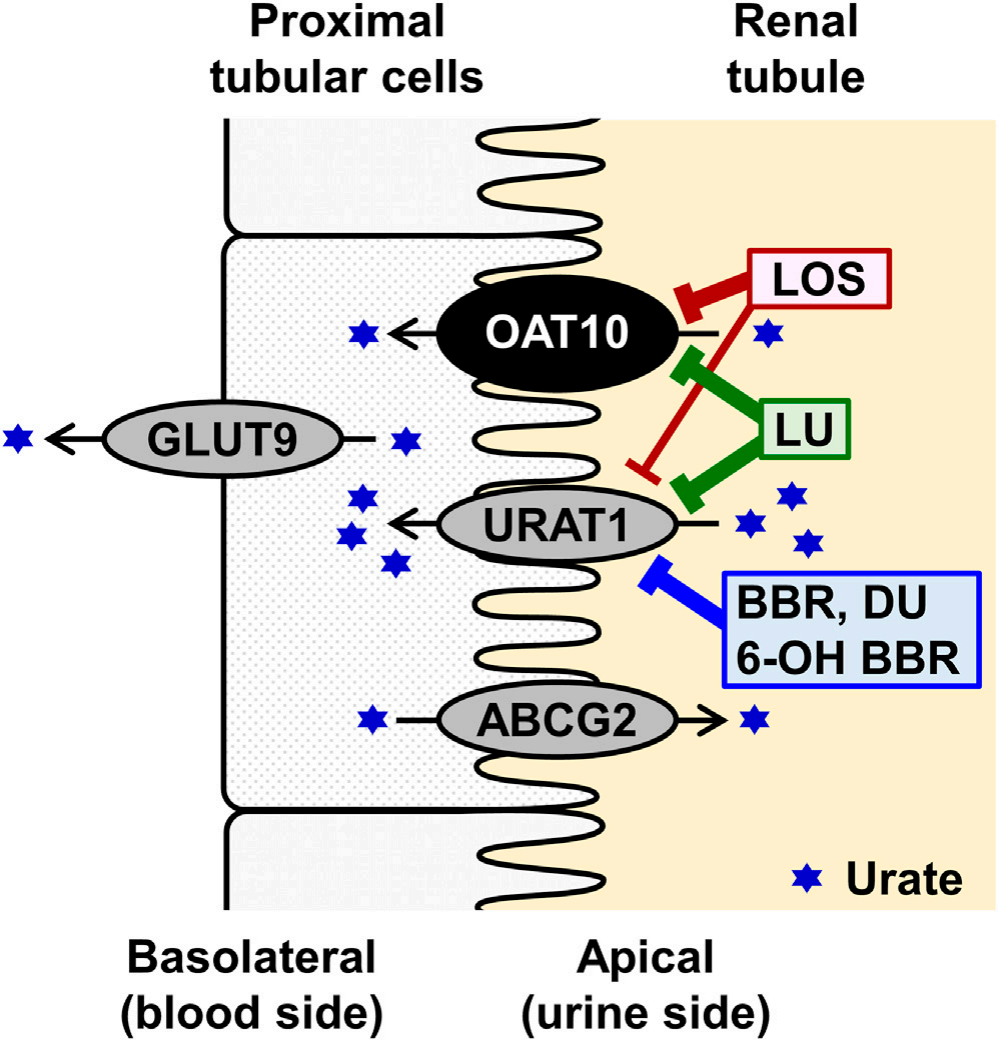
Schematic illustration of OAT10 function as a renal urate re-absorber. The results of interaction with uricosuric compounds and their abbreviations are shown in Figure 5 and its legend, respectively.

First, we conducted inhibitory tests in the presence of each authentic compound at the maximum soluble levels in the transport buffer (Table 2). The results revealed that, contrary to all the urate synthesis inhibitors and fenofibrate, five compounds—benzbromarone, 6-hydroxybenzbromarone (an active metabolite of benzbromarone), dotinurad, lesinurad, and losartan—decreased the urate transport activity of OAT10 to < 20% that of the vehicle control (Figure 5A). Next, we compared their effects at 10 μM on the urate transport activity of OAT10 and URAT1 (Figure 5B), which demonstrated that i) benzbromarone, 6-hydroxybenzbromarone, and dotinurad were more specific to URAT1 than OAT10; ii) lesinurad inhibited OAT10 and URAT1 comparably; iii) losartan exhibited a stronger inhibitory effect on OAT10 than URAT1. These results were confirmed by further investigations of concentration-dependent inhibitory effects and calculated IC_50_ values (Figure 5C). Given the proposed mechanism, lesinurad (Miner et al., 2016) and losartan (Lee and Terkeltaub, 2006) do and can inhibit URAT1 in the kidney, respectively; hence, they may also act as renal OAT10 inhibitors in clinical situations (Figure 6). Of note, given that lesinurad reportedly inhibits not only URAT1 but also other transporters such as OATP1B1, OCT1, OAT1 and OAT3 *in vitro* (Garg et al., 2018), lesinurad is not necessarily highly-specific to URAT1. Thus, the OAT10-inhibitory effect of lesinurad we identified here is not inconsistent with previous findings.

## Discussion

In this study, we investigated the effect of *OAT10* c.1129C>T on renal urate handling in a Japanese male population. The results, including immunohistochemical analyses, demonstrated that the dysfunction of OAT10 leads to an increase in the net activities of both renal urate secretion and a decrease in serum urate levels (Figure 1), which is consistent with our working hypothesis. With the urate-lowering effect of *OAT10* c.1129C>T*,* β (coefficient for the effect allele, 1129T) was −0.135 mg/dl/allele (Supplementary Table S2). Regarding *ABCG2* c.421C>A (p.Q141K) that is a well-established gout/hyperuricemia risk allele in the physiologically important urate transporter (Matsuo et al., 2009; Woodward et al., 2009), the calculated β for serum urate in the Japanese males (carriers with *ABCG2* 421C/A vs*.* 421C/C) was 0.20 mg/dl/allele in a previous report (Matsuo et al., 2009). Given these pieces of information, together with the protective effect of *OAT10* c.1129C>T on gout susceptibility (Higashino et al., 2020), our data support the physiological importance of OAT10 as a urate transporter in the kidney, although the β of *OAT10* was not so large compared with cases of causal genes for renal hypouricemia (which means that OAT10 has a smaller contribution to renal urate re-uptake than URAT1 and GLUT9). Moreover, functional analyses revealed that OAT10 has a high affinity for urate transport following that of URAT1 (Figure 4; Table 3); we identified lesinurad and losartan as clinically available potential inhibitors of OAT10 (Figure 5). These findings deepen our understanding of the physiological and pharmacological impact of OAT10.

While the function of OAT10 as a urate transporter was reported previously (Bahn et al., 2008; Higashino et al., 2020), the affinity of OAT10 to urate has been poorly studied. With this matter, our kinetic analyses revealed that OAT10 has relatively strong affinity for urate [*K*_m_ (95% confidence interval), 558 (409–804) μM] among already-characterized urate transporters in humans (Table 3). A previous *in vitro* study using *Xenopus* oocytes showed that urate inhibited OAT10-mediated nicotinate transport in a concentration dependent manner with the IC_50_ value of 759 ± 501 μM (Bahn et al., 2008) which was the only published data evaluating the concentration-dependent effect of urate on the OAT10 function. Given these similar values, although little information is available, urate and nicotinate might share an internal tunnel for membrane transport in OAT10 protein. Additionally, based on reported *K*_m_ values for nicotinate of OAT10 (<100 μM, lower than the *K*_m_ value for urate we determined) (Bahn et al., 2008), OAT10 has a higher affinity for nicotinate than urate. Nicotinate (pyridine-3-carboxylate) is smaller than urate (a purine compound), which may make it easier to interact with OAT10.

Considering the protective effect of OAT10 dysfunction on gout susceptibility (OR = 0.67) (Higashino et al., 2020), in the context of the potentially simultaneous inhibition of renal URAT1 and OAT10 (Figure 6), it would be interesting to determine whether lesinurad, which had been an approved uricosuric agent that was recently withdrawn from the market unfortunately because of low market penetration, not because of therapeutic issues (Jansen et al., 2022), could have an anti-gout effect stronger than other uricosuric agents that inhibit only URAT1 in clinical situations. It is possible that the positive effects of OAT10 dysfunction may depend on OAT10 expressed not only in the kidney but also in other tissues; such a notion should be addressed in future clinical studies.

Regarding losartan, we found that its IC_50_ value against OAT10 (2.5 μM) is smaller than that against URAT1 (11.9 μM) (Figure 5). This relationship means that losartan would have a higher affinity to OAT10 than to URAT1; when losartan exists at concentrations enough for URAT1 inhibition, it also inhibits OAT10 in theory. In this context, losartan may have a stronger inhibitory activity towards OAT10 than URAT1 in clinical situations given the similarity of their renal expression as apical transporters (Figure 6), suggesting that the uricosuric effect of losartan may involve the inhibition of both OAT10 and URAT1. The beneficial effect of this angiotensin II receptor blocker, generally used as an antihypertensive drug, has been historically explained by the inhibition of URAT1, but not that of OAT10. Therefore, the stronger inhibitory effect of losartan on OAT10 than URAT1 extends our knowledge of the molecular basis underlying losartan-related uricosuric action.

In contrast to urate synthesis inhibitors, uricosuric agents tended to inhibit OAT10. Regarding the inhibitory potency of benzbromarone, our results are consistent with previous reports wherein high-dose benzbromarone inhibited OAT10 function using a *Xenopus* oocyte system (Bahn et al., 2008; Mandal et al., 2017). However, benzbromarone, 6-hydroxybenzbromarone, and dotinurad, considering already-characterized pharmacokinetic information including unbound plasma maximum concentrations (Supplementary Table S6) (Miyata et al., 2016; Taniguchi et al., 2019) and urine levels, would be selective inhibitors of URAT1 in clinical situations. Hence, combination therapy or improvement of chemical structures to enhance the OAT10 inhibitory activity of such urate-lowering drugs may be a potential strategy to provide a more effective anti-gout (urate-lowering) therapy.

The limitations of this study include the lack of pharmacological evaluations in human bodies regarding the effects of OAT10 inhibition by potentially influential drugs that we proposed above. It will be interesting to examine whether existing drugs that unexpectedly affect serum urate levels can interact with OAT10 or not. Moreover, whereas this *OAT10* variant (c.1129C>T; p.R377C) is common in the Japanese, it is very rare in other populations (Higashino et al., 2020); this should be a reason why previous single nucleotide polymorphism-based approaches (i.e., genome-wide association studies) including meta-analyses (Major et al., 2018; Tin et al., 2019) could not find *OAT10* as a gene associated with serum urate levels. Therefore, when other common and/or rare dysfunctional variants of *OAT10* are found in other populations, further replication studies on OAT10-mediated renal urate handling in humans will be required. Last, as this study is restricted in males, it will be a next issue to investigate whether there could be gender differences in the effects of genetic dysfunction of *OAT10* on serum urate levels and renal urate handling. With this matter, a previous study proposed the presence of a gender difference in the renal expression of OAT10 based on immunoblotting data showing stronger signal in a female rat than in a male rat while the data lacked a loading control for the comparison of rat Oat10 protein levels (Bahn et al., 2008). Of note, there are reportedly intersexual differences in urate handling—age-fertile women show lower and higher levels of serum urate and renal urate excretion than men of similar ages, respectively (Mateos Antón et al., 1986; Stiburkova and Bleyer, 2012); however, post-menopausal women exhibit higher serum urate levels than pre-menopausal women (Wingrove et al., 1998; Hak and Choi, 2008). Based on these complicacies, the potential gender differences should be carefully evaluated in the near future.

Before closing, some future directions of this study are warrant mention. First, given the luminal expression of OAT10 in the renal proximal tubules (Figure 2), detailed histological studies investigating the segmental distribution of OAT10 will be a next step because renal proximal tubules are separated into S1–S3 segments that have reportedly different roles in renal urate handling (Maesaka and Fishbane, 1998). According to a recent transcriptome study that examined renal tubule segments microdissected from murine kidneys (Chen et al., 2021), Oat10 was highly expressed in the S2 and S3 segments than other renal tubule segments; a similar expression pattern was observed in Urat1. To deepen our understanding, confirmation of segmental distribution of each protein in the kidney of mice and humans will be required. Second, addressing how OAT10 and URAT1 can physiologically cooperate/interact with each other will be a future topic. Given their reverse chloride-dependency, when URAT1 function could be cis-inhibited by the (locally) elevated urine Cl^−^, OAT10-mediated urate transport might be enhanced, conversely. Additionally, in patients with renal hypouricemia, extremely high FE_UA_ (≥100%) is typical in renal hypouricemia type 2 (characterized by homozygous of non-functional *GLUT9*) but not in renal hypouricemia type 1 (characterized by homozygous of non-functional *URAT1*; typical FE_UA_, tens of %) (Kawamura et al., 2021). This difference can support the presence of a latent alternative that contribute to renal urate re-uptake in the absence of URAT1 function. This context suggests that OAT10 may be a compensator of URAT1 in certain conditions.

In conclusion, we revealed that the dysfunction of OAT10 enhances renal urate excretion evidenced by both the increase in FE_UA_ and the decrease in serum urate. This means that OAT10 acts as a renal urate re-absorber which could be a molecular target for urate-lowering therapy. Our findings provide a better understanding of renal urate handling systems, paving the way for more effective therapeutic strategies for urate-related diseases including gout and hyperuricemia as modulating renal functions is an important approach for the treatment of life-style related diseases.

## Data Availability

The original contributions presented in the study are included in the article/Supplementary Material, further inquiries can be directed to the corresponding authors.

## Abbreviations

ABCG2, ATP-binding cassette transporter G2; BSA, bovine serum albumin; DMSO, dimethyl sulfoxide; FE_UA_, fractional excretion of uric acid; GLUT9, glucose transporter 9; HBSS, Hanks’ Balanced Salt Solution; IC_50_, half maximal inhibitory concentration; MDCKII, Madin-Darby canine kidney II; *V*_max_, maximal velocity; *K*_m_, Michaelis-Menten constant; OR, odds ratio; OAT10, organic anion transporter 10; QTL, quantitative trait locus; URAT1, urate transporter 1.
