# Supplementary material for: OAT10/SLC22A13 Acts as a Renal Urate Re-Absorber: Clinico-Genetic and Functional Analyses With Pharmacological Impacts
Source: Front Pharmacol. 2022 Apr 6;13:842717. doi: 10.3389/fphar.2022.842717 (PMC9019507; doi:10.3389/fphar.2022.842717)
Supplement: Supplementary file 1 [file DataSheet1.pdf]

Supplementary Material

1 Supplementary Figures

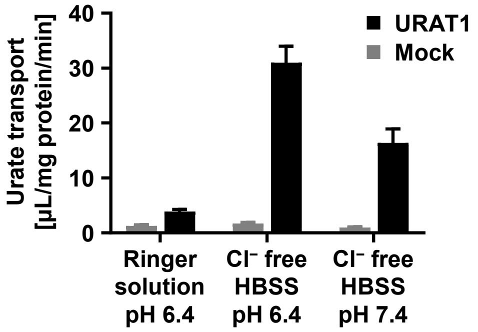

Supplementary Figure S1 | Effects of the differences in transport buffer composition on URAT1-mediated urate transport. Transiently URAT1-expressing 293A cells were subjected to a cell-based urate transport assay, 48 h after transfection. As a transport buffer, Ringer solution, which contains Cl^-, or Cl^--free Hanks' Balanced Salt Solution (HBSS) containing 10 µM [8-^{14}C]-urate was used. In Cl^--free HBSS (pH 6.4), higher URAT1-dependent urate transport activity was detected rather than in Ringer solution (pH 6.4). Acidic pH-dependent urate transport activities of URAT1 in Cl^--free HBSS were found. Data are expressed as the mean ± SD; *n* = 4.

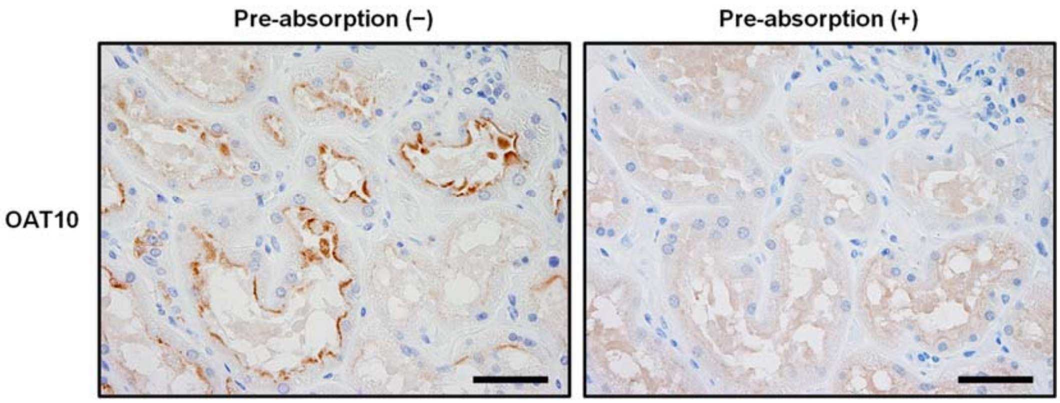

Supplementary Figure S2 | Immunohistochemical detection of OAT10 on the luminal membranes of the renal proximal tubules verified by antigen absorption test. Serial sections of the normal part of the human kidney obtained from a male patient with renal cell carcinoma were analyzed with the anti-OAT10 antibody (1:20 diluted) that was generated in this study. *Left*, stained positively with the anti-OAT10 antibody; *right*, loss of luminal staining following pre-incubation of the primary antibody with OAT10 peptides. These data demonstrate the specificity of the anti-OAT10 antibody. Bars, 50 µm.

2 Supplementary Tables

Supplementary Table S1 | Key resources.

REAGENT or RESOURCE	SOURCE	IDENTIFIER
Antibodies		
Rabbit polyclonal anti-OAT10	Generated for this paper	
Dako EnVision^{TM} Kit/HRP	Dako	Cat# K1491
Rabbit polyclonal anti-EGFP	Life technologies	Cat# A11122; RRID: AB_221569
Rabbit polyclonal anti-α-tubulin	Abcam	Cat# ab15246; RRID: AB_301787
Donkey anti-rabbit IgG-horseradish peroxidase (HRP)-conjugate	GE Healthcare	Cat# NA934V; RRID: AB_772206
(3F2/D8) Podocalyxin/gp135	Developmental Studies Hybridoma Bank	Cat# 3F2/D8; RRID: AB_2618385
Goat anti-Mouse IgG (H+L) Cross-Adsorbed Secondary Antibody, Alexa Fluor 546	Thermo Fisher Scientific	Cat# A-11003; RRID: AB_2534071
Chemicals		
[8-^{14}C]-Uric acid (53 mCi/mmol)	American Radiolabeled Chemicals	Cat# ARC0513
Uric Acid	FUJIFILM Wako Pure Chemical	Cat# 210-00225; CAS: 69-93-2
Clear-sol II	Nacalai Tesque	Cat# 09136-83
Dimethyl Sulfoxide	Nacalai Tesque	Cat# 13445-74; CAS: 67-68-5
Polyethelenimine “MAX” (PEI-MAX)	Polysciences	Cat# 24765; CAS: 49553-93-7
6-Hydroxybenzbromarone	Alsachim	Cat# C7859; CAS: 152831-00-0
Allopurinol	FUJIFILM Wako Pure Chemical	Cat# 019-12502; CAS: 315-30-0
Benzbromarone	FUJIFILM Wako Pure Chemical	Cat# 028-15851; CAS: 3562-84-3
Dotinurad	Kindly provided from Fuji Yakuhin	FYU-981; CAS: 1285572-51-1
Febuxostat	Tokyo Chemical Industry	Cat# F0847; CAS: 144060-53-7
Fenofibrate	Sigma-Aldrich	Cat# F6020; CAS: 49562-28-9
Lesinurad	Sigma-Aldrich	Cat# SML1607; CAS: 878672-00-5
Losartan potassium	LKT laboratories	Cat# L5873; CAS: 124750-99-8
Oxypurinol	FUJIFILM Wako Pure Chemical	Cat# 151-02761; CAS: 2465-59-0
Probenecid	Sigma-Aldrich	Cat# P8761; CAS: 57-66-9
Topiroxostat	MedChemExpress	Cat# HY-14874; CAS: 577778-58-6
Critical Commercial Assays		
Pierce^{TM} BCA Protein Assay Reagent A	Thermo Fisher Scientific	Cat# 23223
Pierce^{TM} BCA Protein Assay Reagent B	Thermo Fisher Scientific	Cat# 23224
PureLink™ HiPure Plasmid Filter Midiprep Kit	Thermo Fisher Scientific	Cat# K210015
Recombinant DNA		
The complete human OAT10 cDNA	Higashino *et al.*, 2020 (1)	NCBI Ref Sequence: NM_004256
The complete human URAT1 cDNA	Toyoda *et al.*, 2020 (2)	NCBI Ref Sequence: NM_144585.3
Experimental Models: Cell Lines		
293A	Invitrogen	R70507
MDCKII	Toyoda *et al.*, 2016 (3)	N/A
Software and Algorithms		
Excel 2019	Microsoft	https://www.microsoft.com/ja-jp/
GraphPad Prism 8	GraphPad Software	https://www.graphpad.com/

N/A, not available.

Supplementary Table S2 | Association between a dysfunctional variant of *OAT10*/*SLC22A13*, rs117371763 (c.1129C>T, p.R377C) and serum urate levels in a Japanese male population (1129C/C vs. 1129C/T).

	Genotypes at c.1129C>T		*P* value	β	95%CI
	C/C	C/T			
Serum urate [mg/dL]	6.04 ± 0.02	5.90 ± 0.06	3.52×10^{-2}	−0.135	−0.260 – −0.009
Number	4,128	384			

Data are expressed as mean ± SEM.

CI, confidence interval.

Supplementary Table S3 | Association between a dysfunctional variant of *OAT10*/*SLC22A13*, rs117371763 (c.1129C>T, p.R377C) and FE_{UA} in a Japanese male population (1129C/C vs. 1129C/T).

	Genotypes at c.1129C>T		*P* value	β	95%CI
	C/C	C/T			
FE_{UA} [%]	4.02 ± 0.06	4.63 ± 0.20	1.91×10^{-3}	0.611	0.226 – 0.997
Number	1,369	143			

Data are expressed as mean ± SEM.

CI, confidence interval; FE_{UA}, fractional excretion of uric acid.

Supplementary Table S4 | Association between a dysfunctional variant of *OAT10*/*SLC22A13*, rs117371763 (c.1129C>T, p.R377C) and serum urate levels in a Japanese male population (1129C/C vs. 1129C/T + 1129T/T).

	Genotypes at c.1129C>T		*P* value	β	95%CI
	C/C	C/T + T/T			
Serum urate [mg/dL]	6.04 ± 0.02	5.90 ± 0.06	2.31×10^{-2}	−0.144	−0.268 – −0.020
Number	4,128	393			

Data are expressed as mean ± SEM.

CI, confidence interval.

Supplementary Table S5 | Association between a dysfunctional variant of *OAT10*/*SLC22A13*, rs117371763 (c.1129C>T, p.R377C) and FE_{UA} in a Japanese male population (1129C/C vs. 1129C/T + 1129T/T).

	Genotypes at c.1129C>T		*P* value	β	95%CI
	C/C	C/T + T/T			
FE_{UA} [%]	4.02 ± 0.06	4.57 ± 0.20	4.28×10^{-3}	0.552	0.174 – 0.930
Number	1,369	149			

Data are expressed as mean ± SEM.

CI, confidence interval; FE_{UA}, fractional excretion of uric acid.

Supplementary Table S6 | Interaction scores to estimate the possible inhibition of OAT10 and URAT1 by each tested compound based on plasma concentrations. As an interaction score, we determined the value of f_uC_{max}/IC_{50}; the large value of this score reflects the possibility of inhibition by tested compounds in humans at clinical dose.

**Compounds**	**f_u** [*]	**C_{max} [μM]** [*]	**f_uC_{max} [μM]**	**with OAT10**		**with URAT1**	
				**IC_{50} [μM]**	**Interaction scores [f_uC_{max}/IC_{50}]**	**IC_{50} [μM]**	**Interaction scores [f_uC_{max}/IC_{50}]**
Benzbromarone	0.037	5.4	2.0×10^{-1}	37.2	5.4×10^{-3}	0.817	0.25
6-Hydroxybenzbromarone	N/A	N/A	N/A	15.9	N/A	0.477	N/A
Dotinurad	0.007	1.2	8.4×10^{-3}	7.7	1.1×10^{-3}	0.043	0.20
Lesinurad	0.019	78.8	1.5	13.6	0.11	14.6	0.10
Losartan	0.020	1.4	2.8×10^{-2}	2.5	1.1×10^{-2}	11.9	2.4×10^{-3}

[*] These values are from previous studies (4, 5). IC_{50}, the half-maximal inhibitory concentration values of target compounds against urate transport by OAT10 or URAT1; f_u, the fraction of drug unbound in human plasma; C_{max}, maximum concentration in human plasma; f_uC_{max}, maximum unbound concentration in human plasma; N/A, not available.

3 Supplementary References

1. Higashino T, Morimoto K, Nakaoka H, Toyoda Y, Kawamura Y, Shimizu S, et al. Dysfunctional missense variant of OAT10/SLC22A13 decreases gout risk and serum uric acid levels. *Ann Rheum Dis* (2020) 79(1):164-6. doi: 10.1136/annrheumdis-2019-216044. PubMed PMID: 31780526; PubMed Central PMCID: PMC6937405.
2. Toyoda Y, Takada T, Saito H, Hirata H, Ota-Kontani A, Kobayashi N, et al. Inhibitory effect of Citrus flavonoids on the in vitro transport activity of human urate transporter 1 (URAT1/SLC22A12), a renal re-absorber of urate. *NPJ Sci Food* (2020) 4:3. doi: 10.1038/s41538-020-0063-7. PubMed PMID: 32047858; PubMed Central PMCID: PMC7002704.
3. Toyoda Y, Takada T, Miyata H, Ishikawa T, Suzuki H. Regulation of the Axillary Osmidrosis-Associated ABCC11 Protein Stability by N-Linked Glycosylation: Effect of Glucose Condition. *PLoS One* (2016) 11(6):e0157172. doi: 10.1371/journal.pone.0157172. PubMed PMID: 27281343; PubMed Central PMCID: PMC4900533.
4. Taniguchi T, Ashizawa N, Matsumoto K, Saito R, Motoki K, Sakai M, et al. Pharmacological Evaluation of Dotinurad, a Selective Urate Reabsorption Inhibitor. *J Pharmacol Exp Ther* (2019) 371(1):162-70. doi: 10.1124/jpet.119.259341. PubMed PMID: 31371478.
5. Miyata H, Takada T, Toyoda Y, Matsuo H, Ichida K, Suzuki H. Identification of Febuxostat as a New Strong ABCG2 Inhibitor: Potential Applications and Risks in Clinical Situations. *Front Pharmacol* (2016) 7:518. doi: 10.3389/fphar.2016.00518. PubMed PMID: 28082903; PubMed Central PMCID: PMC5187494.
